# A Short Review of Iron Metabolism and Pathophysiology of Iron Disorders

**DOI:** 10.3390/medicines6030085

**Published:** 2019-08-05

**Authors:** Andronicos Yiannikourides, Gladys O. Latunde-Dada

**Affiliations:** 1 Faculty of Life Sciences and Medicine, Henriette Raphael House Guy’s Campus King’s College London, London SE1 1UL, UK; 2 Department of Nutritional Sciences, School of Life Course Sciences, King’s College London, Franklin-Wilkins-Building, 150 Stamford Street, London SE1 9NH, UK

**Keywords:** Iron, metabolism, anemia, hepcidin, disorders

## Abstract

Iron is a vital trace element for humans, as it plays a crucial role in oxygen transport, oxidative metabolism, cellular proliferation, and many catalytic reactions. To be beneficial, the amount of iron in the human body needs to be maintained within the ideal range. Iron metabolism is one of the most complex processes involving many organs and tissues, the interaction of which is critical for iron homeostasis. No active mechanism for iron excretion exists. Therefore, the amount of iron absorbed by the intestine is tightly controlled to balance the daily losses. The bone marrow is the prime iron consumer in the body, being the site for erythropoiesis, while the reticuloendothelial system is responsible for iron recycling through erythrocyte phagocytosis. The liver has important synthetic, storing, and regulatory functions in iron homeostasis. Among the numerous proteins involved in iron metabolism, hepcidin is a liver-derived peptide hormone, which is the master regulator of iron metabolism. This hormone acts in many target tissues and regulates systemic iron levels through a negative feedback mechanism. Hepcidin synthesis is controlled by several factors such as iron levels, anaemia, infection, inflammation, and erythropoietic activity. In addition to systemic control, iron balance mechanisms also exist at the cellular level and include the interaction between iron-regulatory proteins and iron-responsive elements. Genetic and acquired diseases of the tissues involved in iron metabolism cause a dysregulation of the iron cycle. Consequently, iron deficiency or excess can result, both of which have detrimental effects on the organism.

## 1. Introduction

Iron is the second most abundant metal on earth, comprising about 5% of the earth’s crust [1]. Its significance to humans is paramount, as it is a vital micronutrient for human existence. Being a d-block transition metal, it interchanges between various oxidation states, which empowers it to participate in electron transfer and also bind to several biological ligands. The two most common iron states are the divalent ferrous (Fe^2+^) and the trivalent ferric (Fe^3+^). Within the human body, iron is required as a cofactor for many haemoproteins and non-haem iron-containing proteins. Haemoproteins include haemoglobin and myoglobin that are responsible for oxygen binding and transport, catalase and peroxidase enzymes, which take part in oxygen metabolism, and cytochromes, which are involved in electron transport and mitochondrial respiration. Non-haem iron-containing proteins also have crucial functions, as these are used in DNA synthesis, cell proliferation and differentiation, gene regulation, drug metabolism, and steroid synthesis [1].

The total amount of iron in a 70 kg man is about 3500–4000 mg, corresponding to an average concentration of 50–60 mg of iron per kg of body weight. The vast majority (2300 mg, 65%) of iron in the body is found in the haemoglobin of erythrocytes. About a tenth of total iron (350 mg) is present in the myoglobin of muscle and enzymes and cytochromes of other tissues. Of the remainder, approximately 500 mg is found in macrophages of the reticuloendothelial system (RES), about 200–1000 mg is stored in hepatocytes in the form of ferritin, while 150 mg of iron is found in the bone marrow [2,3].

**Figure 1:**
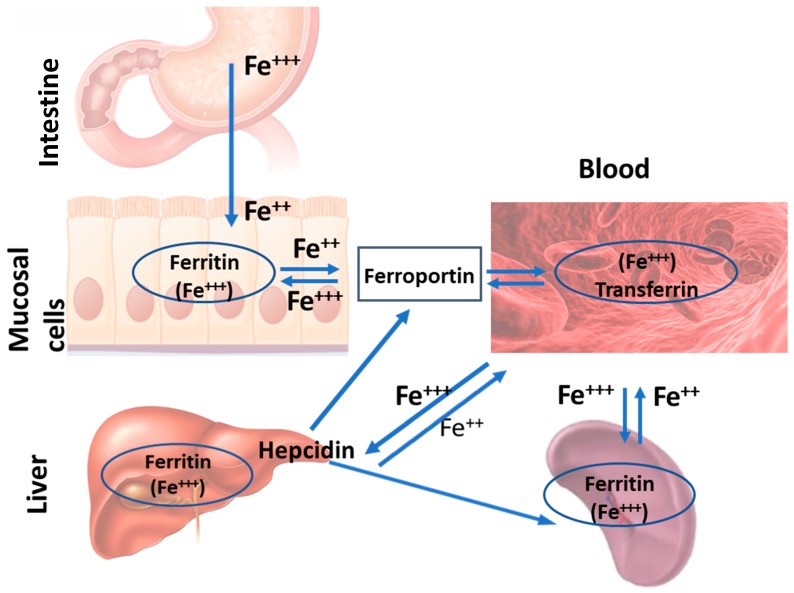
The main tissues involved in the regulation of systemic iron metabolism. Duodenal enterocytes are responsible for dietary iron absorption. Upon absorption, iron circulates around the body bound to the protein transferrin and is taken up by different tissues for utilisation. The reticuloendothelial system, which includes the splenic macrophages, recycles iron from senescent erythrocytes. Among many other functions, the liver produces the hormone hepcidin. Hepcidin controls the release of iron from enterocytes and macrophages into the circulation and is regarded as the master regulator of systemic iron metabolism.

The duodenum plays a very significant role in dietary iron absorption. The absorbed iron can be stored in the enterocytes or enter the circulation and be transported around the body bound to the liver-derived plasma protein transferrin (Tf). It is then taken up by tissues and utilised for many processes, such as erythropoiesis in the bone marrow, myoglobin synthesis in muscle, and oxidative metabolism in all respiring cells. Splenic, hepatic, and bone marrow macrophages, which belong to the RES, have the task of recycling iron from senescent erythrocytes. The liver has an important storing and regulatory function. Through the production of the hormone hepcidin, it controls the release of iron from enterocytes and macrophages into the circulation. Therefore, this permits the fine regulation and the maintenance of plasma iron concentrations within physiological levels. Figure 1 presents the main tissues where iron metabolism occurs.

Approximately 1–2 mg of iron is lost daily from the body through enterocyte and skin desquamation and through haemorrhages and parasitic infestations. No active mechanism of iron excretion exists. Consequently, a daily quantity of 1–2 mg of intestinal iron absorption is required for iron homeostasis. This demand is increased in physiological conditions such as growth, pregnancy, and menstruation. Meanwhile, about 25 mg of iron is recycled every day by the RES as senescent erythrocytes are phagocytosed. This means most of human iron homeostasis is dependent on iron recycling [4]. Tight regulation of iron metabolism is imperative for its homeostasis.

This review assesses the roles of the intestine, the bone marrow, the spleen, and the liver in iron metabolism. Furthermore, a consideration of tissue-specific pathological situations and their effects on iron homeostasis is presented. Finally, management of these iron-associated disorders is included.

## 2. Intestine

**Figure 2:**
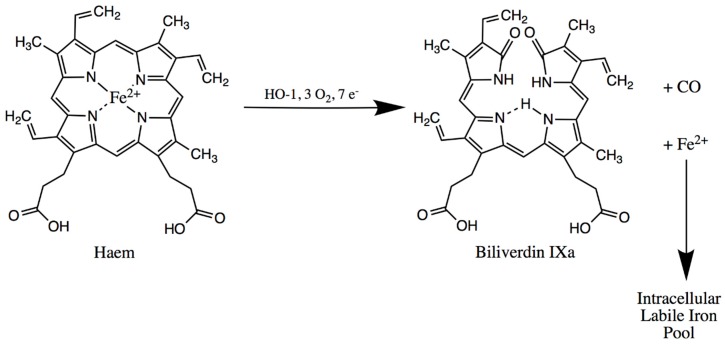
In the enterocytes, haem can be degraded to free iron, which enters the intracellular labile iron pool.

An average western-world diet includes the ingestion of about 15–20 mg of iron, 10% of which is present in the haem form and the remainder in the non-haem/ionic form. Only about a tenth of the consumed iron is absorbed, predominantly within the duodenum and lesser amounts in the jejunum [2]. Most of the ingested haem-iron is found in haemoglobin and myoglobin of meat proteins. The low gastric pH releases these proteins from meat, and subsequent protease action in the stomach and the intestine releases free haem [5]. It has been proposed that enterocytes absorb haem via the Haem Carrier Protein 1 transporter (HCP1) localised on their brush-border membrane [6]. Evidence from a later study showed that this protein is a proton-coupled folate transporter (PCFT), and therefore the acronym PCFT/HCP1 is sometimes used for the transporter [7]. Once haem enters the enterocyte, it can be degraded via the action of haem oxygenase (HO-1) to release free iron, which joins the intracellular labile iron pool (LIP), as depicted in Figure 2. Moreover, intact haem may also be absorbed into the circulation via two exporter proteins found on the enterocyte basolateral membrane: the breast cancer resistant protein (BCRP) and the feline leukaemia virus subgroup C (FLVCR) [8].

**Figure 3:**
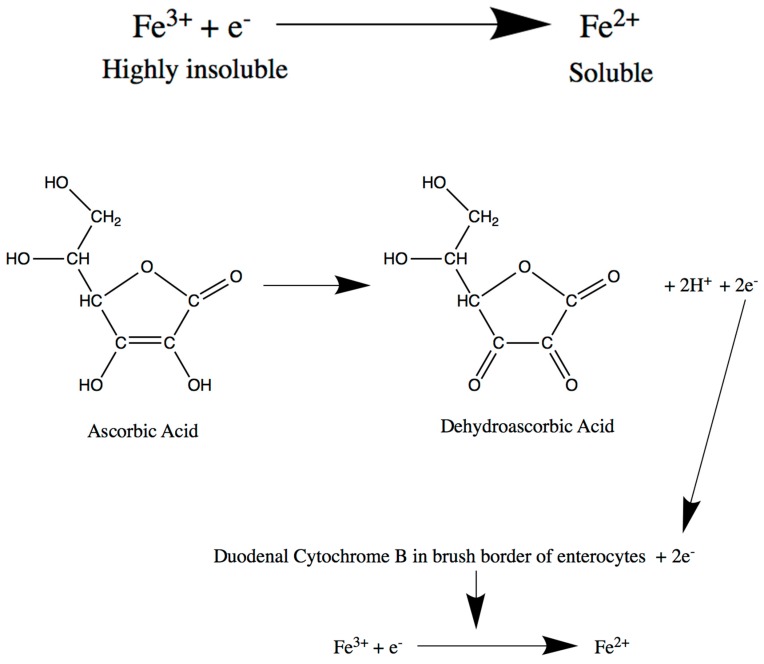
Low stomach pH and dietary ascorbic acid reduces non-haem iron from the highly insoluble Fe^3+^ form to Fe^2+^, which is more readily absorbed. Duodenal cytochrome b accepts electrons intracellularly from oxidation of ascorbic acid into dehydroascorbic acid and uses these to catalyse the reduction of Fe^3+^ to Fe^2+^.

Non-haem iron is found in meat and plant foods and largely exists in the ferric (Fe^3+^) form. Ferric iron, unlike ferrous (Fe^2+^) iron, is highly insoluble and not easily absorbed. Its reduction to ferrous iron is essential, since the latter is the preferred form for absorption. The low pH created in the stomach, as well as the presence of dietary ascorbic acid (vitamin C), reduce Fe^3+^ to Fe^2+^ ions, enhancing solubility and absorption [9]. The relatively more acidic environment in the duodenum and the proximal jejunum compared to more distal intestinal segments explains why iron absorption occurs proximally within the gastrointestinal tract. Very importantly, the duodenal cytochrome b (Dcytb) protein is localised on the brush-border membrane of the enterocytes. Dcytb is a ferrireductase enzyme responsible for the reduction of Fe^3+^ to Fe^2+^ ions [10]. It accepts electrons intracellularly from the oxidation of ascorbic acid into dehydroascorbic acid and uses these to catalyse the reduction of ferric to ferrous iron (Figure 3), thus portraying one way in which ascorbic acid is involved in iron absorption [11]. Following this reduction process, divalent Fe^2+^ ions are transported into the duodenal enterocytes via the divalent metal transporter 1 (DMT1). DMT1 is another duodenal brush-border membrane protein, which transports several divalent metal ions across the membrane, including ferrous iron, zinc(II), and copper(II) [12]. This transport is proton(H^+^)-coupled and depends on the presence of luminal H^+^ ions. This is accomplished by another brush-border membrane transporter, the Sodium/Hydrogen Exchanger (NHE), which allows proton recycling across the duodenal luminal membrane.

Once absorbed, ferrous iron joins the intracellular LIP, and it is postulated that this free and highly reactive ion is chelated by low-molecular-weight organic acids, amino acids, and proteins [9]. When there is a low demand for iron in the body, absorbed iron is stored within the enterocyte in the form of ferritin, an intracellular iron storage protein. Ferritin is made up of heavy and light chain subunits, which create a spherical hollow space that can accommodate up to 4500 iron ions. Enterocytes have a short life expectancy, as they are desquamated and replaced every few days. Consequently, iron stored in ferritin is lost upon enterocyte desquamation [9]. On the contrary, when iron demand is high, the absorbed ferrous iron is transported across the basolateral membrane into blood. This phase is controlled by ferroportin 1 (FPN1), a ferrous iron export protein that modulates how much of the enterocyte iron is absorbed into circulation and made available to the body [13]. For this reason, FPN1 expression is tightly regulated by the hepatic hormone hepcidin.

**Figure 4:**
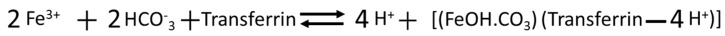
Iron binding to transferrin.

Oxidation of the absorbed ferrous iron back to ferric iron is required for transport by the plasma protein Tf. This is achieved by hephaestin (HEPH), a multi-copper ferroxidase enzyme anchored to the basolateral enterocyte membrane and coupled to FPN1, which catalyses the oxidation of Fe^2+^ to Fe^3+^ ions [14]. Additionally, there is ceruloplasmin, a circulating copper-carrying protein synthesised in the liver, which also exhibits ferroxidase activity [15]. Fe^3+^ then binds to Tf, which has a high affinity for Fe(III) and enables iron transport around the body (Figure 4). Apotransferrin (apo-Tf) is the unbound form of this transporter and contains two ferric binding sites, of which none, one, or both may be filled. Under physiological conditions, only about 30% of Tf is saturated. This has an important buffering function that prevents any sudden build-up of non-transferrin-bound iron (NTBI), which, being highly reactive, may cause oxidative injury if taken up by the tissues [1].

### Intestine-Related Iron Metabolism Disorders

Several physiological abnormalities of the intestines interfere with iron metabolism. Some are related to increased iron loss from the gastrointestinal tract. Increased gastrointestinal bleeding creates an iron deficit through blood loss and leads to iron-deficiency anaemia. Intestinal haemorrhage can be a result of gastric or duodenal ulcers eroding into blood vessels or due to the presence of a gastric cancer. Inflammatory bowel disease also causes bleeding and subsequent iron loss. In the distal segment of the alimentary tract, haemorrhages can arise due to colonic cancer or haemorrhoids. Proximally, oesophageal varices, oesophagitis, and oesophageal cancer are other causes of blood loss. Moreover, hookworm infection, which affects hundreds of millions of people worldwide, is another cause of iron-deficiency anaemia. These worms parasitize the gut for many years, extravasating 0.3–0.5 mL of blood from the intestinal mucosa every day [16].

Iron malabsorption is another broad category of deviations from normal intestinal iron metabolism. This arises from total or partial gastrectomy, where reduced gastric acid production results in a more alkaline luminal environment that does not favour iron absorption. Iron malabsorption can also be a consequence of coeliac disease, in which villous atrophy reduces the intestinal mucosal surface area available for absorption. Similarly, Crohn’s disease is another malabsorption syndrome. Furthermore, infection by helicobacter pylori causes gastric atrophy, which reduces iron absorption [17]. Moreover, this infection can incite gastric and duodenal ulcers with subsequent blood and iron loss. Finally, some drugs, for example, proton-pump inhibitors and anti-acid agents associated with increased pH, interfere with iron absorption by reducing intestinal uptake of the nutrient [18].

## 3. Bone Marrow

As already mentioned, the amount of iron in haemoglobin accounts for about two thirds of the mass of iron in the human body. The bone marrow is thus the prime iron consumer in the body, since this is where erythropoiesis takes place. Almost all nucleated cells in the body express transferrin receptors (TfR1) on their plasma membrane, which have a high affinity for iron-bound transferrin. There is a correlation between TfR1 levels and cellular iron requirements. This receptor is highly expressed in erythroblasts but to a lesser extent in hepatocytes [19]. The receptor is a disulphide-linked transmembrane glycoprotein, which forms a homodimer, and as each of its two subunits can bind one Tf molecule, a total of two Tf molecules can be bound by each TfR1 [20]. Factors such as temperature or energy do not affect the efficiency of the interaction between Tf and TfR1. The factor that affects this interaction is the iron content of the Tf molecule. The affinity of interaction increases with increasing number of Fe^3+^ ions bound to Tf. Therefore, diferric Tf (Fe_2_Tf) has the greatest affinity for TfR1, monoferric Tf (Fe_1_Tf) has an intermediate affinity, while apo-Tf expresses the lowest affinity for TfR1 [1]. An important property of TfR1 is that at an extracellular pH (7.4), when it binds to Fe_2_Tf, it reduces the accessibility of the latter to iron acceptors and consequently diminishes the possibility for nonspecific iron release from Fe_2_Tf at the cell membrane [21].

Transferrin-bound iron (TBI) enters the cell via receptor-mediated endocytosis. Initially, iron-bound transferrin binds to TfR1 at the cell membrane, leading to the production of the iron-transferrin/transferrin receptor (Fe_2_Tf-TfR1) complex [22]. Consequently, energy-dependent endocytosis of Fe_2_Tf-TfR1 complexes occurs as the cell membrane becomes surrounded by clathrin proteins. These proteins cause the invagination of the cell membrane containing the Tf-TfR1 complexes and the formation of clathrin-coated endosomes [23]. Subsequently, clathrin proteins are removed, and the endosomal contents are rendered acidic due to proton influx via an ATP-dependent proton pump at the endosomal membrane. The resultant low pH causes conformational changes in both Tf and TfR1, which promotes the dissociation of iron from the Tf-TfR1 complex. However, apo-Tf still remains bound to TfR1, since at this low pH (≤5.5), apo-Tf binds to TfR1 with the same affinity as Fe_2_Tf does at pH 7.0 [24]. The released Fe^3+^ ions undergo reduction to Fe^2+^ by the ferrireductase STEAP3 (six transmembrane epithelial antigen of prostate) protein, which belongs to the metalloreductase family [25]. Ferrous iron is transported out of the endosome via DMT1 and becomes available for either direct use or storage in ferritin. Finally, the apo-Tf-TfR1 complex is transferred back to the cell membrane, where the extracellular pH (7.4) reduces the affinity of apo-Tf for TfR1, causing dissociation and the release of apo-Tf back into the circulation [24]. Hence, both molecules are recycled. Recent findings reveal evidence that TfR2 mediates lysosomal delivery of transferrin in erythroid progenitors that TfR2 may also contribute to mitochondrial iron delivery [26]. Hence, TfR2 null donor cells manifest microcytic red cells and abnormal hemoglobinization in mouse marrow transplant models [27].

**Figure 5:**
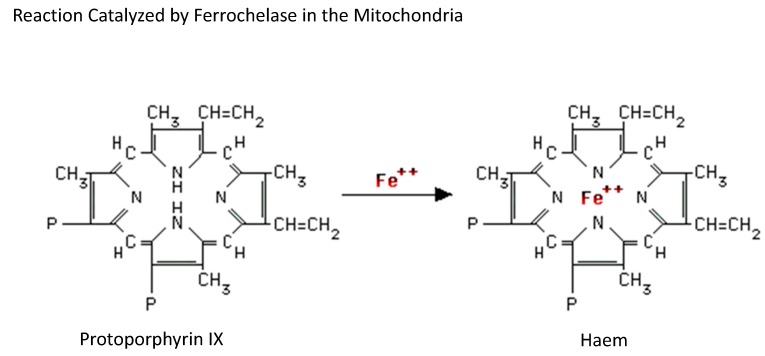
Haem synthesis. Haem synthesis is a complex multistep reaction whose first and final steps occur in the mitochondrion, while the intermediate steps take place in the cytoplasm. Iron is imported into the mitochondrion and inserted into protoporphyrin IX to produce haem. Subsequently, haem is exported into the cytosol, where it combines with α and β globin chains formed on ribosomes to create haemoglobin.

Erythropoiesis begins in the bone marrow once pluripotent stem cells differentiate into erythroid precursor cells in the presence of the hormone erythropoietin. However, haemoglobin synthesis is only initiated when the erythroid precursor cells start to differentiate into erythroblasts, expressing transferrin receptors at this stage and taking up iron. The first and the final steps of haem synthesis occur in the mitochondrion, while the intermediate steps take place in the cytoplasm. The first step, which is essentially rate-limiting, involves the conversion of glycine and succinyl coenzyme A to δ-aminolaevulinic acid (ALA) catalysed by the enzyme ALA synthase. Pyridoxine (vitamin B_6_) is the coenzyme for this reaction and is stimulated by erythropoietin and inhibited by haem. The laevulinate product is exported into the cytoplasm, and the condensation of two ALA molecules forms a monopyrrole pyrrole ring (porphobilinogen) (PBG) by a condensation reaction. Four molecules of PBG form hydroxymethylbilane (HMB) tetrapyrrole macrocycle, which cyclizes to form uroporphyrinogen III. The next step is the modification of the acetate side chains. The decarboxylation reactions produce coproporphyrinogen, which enters the mitochondrion to be converted into porphyrinogen IX, i.e., the sequential oxidative decarboxylation of the two propionate groups of pyrrole rings to two vinyl groups. The final step in the haem biosynthetic pathway is the oxidation of porphyrinogen IX to protoporphyrin IX (Figure 5). Meanwhile, mitoferrin-1, an iron transporter, facilitates iron entry into the mitochondrion [28]. By interacting with the ATP-binding cassette transporter ABCB10, mitoferrin-1 binds to mitochondrial ferrochelatase to form an oligomeric complex [29]. Ferrochelatase catalyses the final step of this synthetic reaction by inserting iron into protoporphyrin IX to produce haem. Subsequently, haem is exported into the cytosol via mitochondrial haem exporters and combines with globin chains formed on ribosomes to generate haemoglobin. With repeated cellular divisions, the nucleus condenses and is eventually lost, thus resulting in a reticulocyte. Ultimately, the remaining RNA is also lost from the circulating reticulocytes, which mature into erythrocytes.

Irrespective of the cell type, the mitochondrion is the organelle consuming the most iron intracellularly. It is a central player in the regulation of cellular iron metabolism. Most of the iron taken up by a cell is imported into the mitochondrion for the synthesis not only of haem but also of iron-sulphur clusters (ISCs). These are cofactors made up of iron cations and sulphide anions. They bind to scaffold proteins and then get targeted to particular proteins [1]. Iron is indeed a vital cofactor in complexes I–IV of the mitochondrial respiratory chain. On the one hand, complexes I–III are enzymes that contain ISCs, whilst on the other hand, complexes III and IV contain haem iron.

### Bone Marrow-Related Iron Metabolism Disorders

Iron-loading anaemias include a mixture of inherited and acquired anaemias, which are characterized by ineffective erythropoiesis, low hepcidin levels, excessive iron absorption, and secondary iron overload [30]. Abnormal erythropoiesis causes anaemia, leading to an excessive increase in iron absorption and resulting in iron overload. Sideroblastic anaemia, congenital dyserythropoietic anaemias, and low-grade myelodysplastic syndromes are a few examples of iron-loading anaemias due to ineffective erythropoiesis.

In sideroblastic anaemias, hypochromic cells can be seen in the peripheral blood, while excess iron and ring sideroblasts can be observed in the bone marrow, the latter being the diagnostic feature of sideroblastic anaemia. Iron accumulates in the mitochondria of erythroblasts due to abnormal haem synthesis. This creates a ring of iron granules around the nucleus. Sideroblastic anaemia can be inherited as an X-linked disease. One form of this disorder involves a structural defect in the enzyme ALA synthase, which catalyses the first step in haem synthesis. Acquired causes include myelodysplasia, myeloproliferative disorders, myeloid leukaemia, drugs such as isoniazid, alcohol misuse, and lead toxicity. Sideroblastic anaemia can also be secondary to rheumatoid arthritis, carcinomas, and megaloblastic and haemolytic anaemias [8].

## 4. Spleen

As the lifespan of an erythrocyte is approximately 120 days, the bone marrow produces over 200 billion new erythrocytes daily, involving about 24 mg of iron for haem synthesis and erythropoiesis. Daily intestinal iron absorption of 1–2 mg is simply not enough to meet this demand for 24 mg, as this only balances iron loss. Instead, this requirement is mostly met through iron recycling by RES macrophages phagocytosing senescent erythrocytes. Indeed, approximately 80% of plasma iron follows the route from the RES to the bone marrow. The spleen is the major site for iron recycling with some contribution from the liver and the bone marrow. A further function of the RES is the storage of iron during periods of iron excess [31].

Senescent erythrocytes exhibit markers of ageing, such as a decrease in membrane flexibility and the presentation of phosphatidylserine on their cell membrane [32]. These changes are recognised by the splenic macrophages triggering erythrocyte phagocytosis. Following phagocytosis, the erythrocytes are included in a phagolysosome and degraded by hydrolytic enzymes and reactive oxygen species to release their haem. Thereafter, the enzyme HO-1, in the presence of oxygen, catalyses the breakdown of haem into iron(II), carbon monoxide, and biliverdin [33]. Subsequently, Fe^2+^ ions are transported out of the phagolysosome via the natural resistance-associated macrophage protein-1 (NRAMP1) and enter the cytoplasm to join the LIP [34]. NRAMP1 is a hydrophobic protein present only in monocytes and macrophages and has a 64% amino acid sequence identity with DMT1, also known as NRAMP2.

Apart from iron uptake through erythrophagocytosis, the splenic and other macrophages of the RES also obtain iron via other processes. Normally, there is some erythrocyte destruction occurring within the vasculature. This releases haemoglobin into the plasma, which immediately binds to the plasma protein haptoglobin. Upon binding, a haemoglobin–haptoglobin complex is formed, and this inhibits the oxidative activity of haemoglobin. Monocytes and macrophages, and particularly splenic and hepatic ones, exclusively express the CD163 protein receptor. This receptor recognises the haemoglobin–haptoglobin complex and binds to it with high affinity. Then, CD163-mediated endocytosis of the complex takes place, and its degradation follows within the macrophage releasing haemoglobin [35]. In certain cases of augmented haemolysis, such as haemolytic anaemia and thalassaemia, haptoglobin may become saturated with haemoglobin, resulting in free haemoglobin in the circulation. Some of this breaks down to give haem, which then binds to hemopexin, a plasma glycoprotein, to produce a haem–hemopexin complex. A wide range of cell types, including macrophages, possess the low-density lipoprotein receptor-related protein LRP/CD91 that serves as a receptor for the haem–hemopexin complex and causes its endocytosis to further contribute to iron recycling [36]. Finally, macrophages can also obtain iron from bacteria from the circulation via DMT1, as well as TBI via TfR1 [2].

Iron is exported from the macrophage and into the circulation via FPN1, the same iron(II) exporter found in the duodenal enterocyte. Similarly, iron export from macrophages is also subject to regulation by hepcidin, which controls iron recycling. Exported ferrous iron then undergoes oxidation by ceruloplasmin to become ferric iron, which binds to apo-Tf and becomes recirculated. Iron in the cytoplasm of macrophages can also be stored in ferritin. Haemosiderin is an insoluble iron store that results from the partial digestion of ferritin in lysosomes. Under physiological conditions, haemosiderin accounts for a very small percentage of the total body iron quantity and is primarily found in macrophages. As cellular iron content increases, a greater fraction of iron gets deposited into haemosiderin. When there is an excess of iron, this surplus is diverted into haemosiderin and enables the cell to store a greater quantity of iron per unit cellular volume. During iron overload, there is a significant increase in the amount of haemosiderin in macrophages. This has an important safety aspect, as this form of iron storage in macrophages prevents oxidative damage [2,31].

### Spleen-Related Iron Metabolism Disorders

In cases of genetic anaemias and inability of the bone marrow to carry out erythropoiesis, regular blood transfusions may be the only option for treatment. The transfused erythrocytes are phagocytosed by splenic and other RES macrophages, and resultant degradation releases iron, which gets recycled into the bloodstream. With repeated transfusions and erythrophagocytosis, the spleen and other tissues of the RES secrete a very large amount of iron into circulation, leading to a state of iron overload. The iron-binding capacity of Tf becomes saturated, and this results in the presence of free NTBI in the plasma which, through the production of reactive oxygen species, causes organ dysfunction.

Hereditary haemochromatosis and anaemia of chronic disease are two conditions where iron metabolism is disturbed in various tissues, including the spleen and the intestine. Both are related to abnormalities in hepcidin levels, the pathophysiology of which is discussed in greater detail in Section 5. In essence, hepcidin downregulates FPN1 and reduces iron export from tissues such as the RES and the intestine. In hereditary haemochromatosis with low hepcidin levels, there is an upregulation of FPN1, which results in an increased transport of iron into the blood from the RES macrophages and intestinal iron absorption. On the contrary, in anaemia of chronic disease, hepcidin levels are significantly raised. FPN1 downregulation causes reduced intestinal iron absorption, as well as impaired iron release from macrophages of the spleen and liver.

## 5. Liver

The liver has a central and critical role in iron homeostasis, as it synthesises many of the proteins involved in iron metabolism. Above all, the liver is the major site of synthesis of hepcidin, a master regulator of iron homeostasis [37,38]. Hepcidin expression also occurs on a smaller scale in other tissues, such as alveolar macrophages, pancreatic β-cells, and the kidneys [39,40]. Expression of the HAMP gene results in the production of hepcidin preproprotein. This preproprotein undergoes intracellular proteolytic cleavage by the enzyme prohormone convertase furin to yield the active 25 amino-acid-long peptide hormone hepcidin [41].

Hepcidin is the main negative regulator of iron homeostasis. It acts by binding to FPN1 to induce its ubiquitination. Subsequently, the internalisation and lysosomal degradation of FPN1 takes place [42,43]. This down-regulation of FPN1 occurs at the basolateral membrane of duodenal enterocytes, as well as from the plasma membrane of RES macrophages, hepatocytes, and the placenta. In the case of the intestine, this means that iron is blocked inside the enterocytes, and thus less can be absorbed into the circulation, while more iron is lost through enterocyte desquamation. With regards to the RES, the iron taken up into the macrophages through erythrophagocytosis stays trapped inside the cells, since its release into the bloodstream by FPN1 is inhibited. Under physiological conditions, the expression of hepcidin is tightly regulated in order to maintain normal body iron levels. In cases of increased iron levels, hepcidin synthesis is augmented in an effort to reduce intestinal iron absorption, as well as to reduce the release of iron from macrophages of the RES into the bloodstream and thus bring iron levels down to normal. In contrast, when there is iron deficiency, hepcidin production decreases, and this results in increased intestinal iron absorption, as well as increased iron release from the RES, which increases the quantity of iron in the circulation. Similarly, in conditions of anaemia and increased erythropoietic activity, hepcidin expression is suppressed, which increases systemic iron levels [1].

Numerous proteins found on the hepatocellular membrane are considered to act as sensors of iron levels and control the quantity of hepcidin synthesis. These include haemojuvelin (HJV), haemochromatosis protein (HFE), TfR1, and transferrin receptor 2 (TfR2). One pathway that regulates HAMP expression and hepcidin production is the HJV–bone morphogenic protein (BMP) axis. On the hepatocellular membrane, BMP receptors (BMPRs) are found, whilst HJV acts as a co-receptor for BMPs. When BMPs bind to the BMPR–HJV complex, the receptor complex becomes activated and induces the phosphorylation of cytosolic small-mothers-against-decapentaplegic proteins (SMADs) 1, 5, and 8 [44]. These can then bind to SMAD4, and the resulting complex enters the nucleus, where it binds to BMP-responsive elements on the HAMP promoter [45]. This results in the transcription of HAMP and subsequent hepcidin synthesis. BMP6 is one of the BMPs involved in this pathway, and its production takes place in non-parenchymal hepatic cells, such as sinusoidal endothelial cells, hepatic stellate cells, and Kupffer macrophage cells. When body iron levels increase, BMP6 synthesis in the non-parenchymal hepatic cells increases [46]. Consequently, more BMP6 binds to the BMPR-HJV complex, and more hepcidin is synthesised in the hepatocytes.

Apart from the HJV–BMP axis pathway, a different pathway for the regulation of hepcidin synthesis is thought to exist, and this includes the HFE and the TfR2 proteins. Normally, HFE is bound to TfR1 on the hepatocellular membrane [47] at a binding site overlapping with that for Fe_2_Tf. TfR1 expresses a greater affinity for Fe_2_Tf than HFE, whilst its affinity for HFE is higher than for apo-Tf. As iron levels in the blood increase, the quantity of Fe_2_Tf increases. As more Fe_2_Tf binds to TfR1, HFE becomes displaced from TfR1 and associates with TfR2. The Fe_2_Tf–TfR2–HFE complex then induces HAMP expression via the extracellular signal-regulated kinase/mitogen-activated protein kinase (ERK/MAPK) pathway [48]. Some studies suggest that HFE and TfR2 may act independently of each other in regulating hepcidin synthesis [49,50]. Nonetheless, HFE has been shown to interact with BMPR ALK3. This interaction inhibits ALK3 ubiquitination and proteasomal degradation and favours its stabilisation on the hepatocellular membrane. Moreover, HFE increases ALK3 cell-surface expression. All these demonstrate that HFE can regulate hepcidin synthesis via the BMP pathway [51].

**Figure 6:**
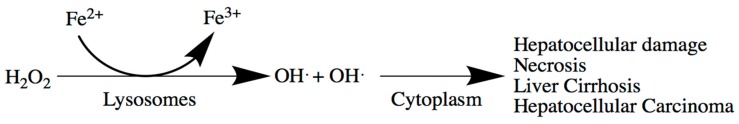
Fenton reaction to produce hydroxyl radicals, which can cause membrane damage and hepatocellular damage, necrosis, liver cirrhosis, or hepatocellular carcinoma once they are in the cytoplasm.

Infection and inflammation trigger an increase in hepcidin synthesis, which serves an important protective function. The reduction in iron levels reduces the growth of iron-dependent micro-organisms and helps to eliminate the infection more rapidly. Exposure of the immune system to pathogens stimulates the release of pro-inflammatory cytokines. One of these is interleukin-6 (IL-6). IL-6 is recognised by receptors on the hepatocellular membrane, and this interaction activates Janus kinases, which phosphorylate signal transducers and activators of transcription (STAT) proteins, mainly STAT3. Phosphorylated STAT3 then enters the nucleus, where it binds to HAMP promoter to stimulate HAMP transcription and subsequent hepcidin production [52]. In the short term, this inflammatory response is beneficial to the organism. However, when inflammation persists, the results can be detrimental. In conditions such as chronic infections, inflammatory diseases, and some malignancies, hepcidin levels remain constantly elevated. This causes a prolonged reduction in intestinal iron absorption, as well as sequestration of iron in RES macrophages. Moreover, in response to inflammation, macrophages synthesise small amounts of hepcidin, which targets FPN1 in an autocrine mode, promoting iron retention within the macrophages [53]. Therefore, less iron is available for bone marrow utilisation, resulting in anaemia of chronic disease. Detailed descriptions of the regulatory mechanisms of hepcidin gene expression in the hepatocyte are reviewed elsewhere [54]. The liver is also an important iron storing organ. It takes up iron from the circulation in the form of TBI via TfR1 and TfR2, NTBI, ferritin, haemoglobin–haptoglobin complexes, and haem–haemopexin complexes for storage. Hepatocytes such as duodenal enterocytes and RES macrophages also possess FPN1 on their plasma membrane, which enables iron efflux from the cell [2]. When there is iron homeostasis, excess iron is stored in hepatocytes in the form of ferritin; however, when iron overload occurs, more iron is stored as haemosiderin. In the case of persistent iron overload, high concentrations of ferritin and haemosiderin accumulate. Lysosomal degradation of ferritin and haemosiderin with reduction of Fe^3+^ produces a high lysosomal Fe^2+^ concentration. Hydrogen peroxide, which is physiologically produced in the cell, is able to pass through biological membranes. It can also pass into the lysosomal compartment, where, if a high Fe^2+^ concentration exists, it will be metabolised via Fenton’s reaction to produce a large quantity of extremely reactive hydroxyl radicals [55]. These radicals can cause lysosomal membrane rupture and, upon their release into the cytoplasm, they can cause hepatocellular damage and necrosis (Figure 6). Consequently, in persistent iron overload states, the liver, being a major iron store, is under the risk of cirrhosis or hepatocellular carcinoma in the longer term [56].

Cellular iron balance mechanisms also exist for the control of the stability and the translation of specific iron-related messenger ribonucleic acids (mRNAs) that code for ferritin, transferrin, TfR1, DMT1, and ALA synthase. Cytoplasmic iron-regulatory proteins (IRPs) bind to specific iron-responsive elements (IREs) on these mRNAs and determine whether these undergo translation or not. IRP–IRE interaction promotes the stability and the translation of the mRNAs for transferrin, TfR1, DMT1, and ALA synthase. However, in the case of ferritin mRNA, IRP–IRE interaction inhibits its translation and ferritin synthesis. In iron deficiency, IRPs bind to target mRNA-IREs, and this interaction increases the synthesis of all the aforementioned proteins, except ferritin, whose synthesis is decreased. This leads to increased intestinal iron absorption via DMT1. Moreover, the increase in transferrin and TfR1 promotes cellular uptake of iron from the circulation, while the decrease in ferritin prevents iron storage. Overall, these changes enable increased cellular utilisation of iron. In contrast, when there is excess cellular iron, the binding capacity of IRPs for IREs is lost. This causes mRNA degradation and diminished synthesis of the above-mentioned proteins, although ferritin-mRNA stabilisation and enhanced ferritin production occur. Consequently, this reduces intestinal iron absorption, decreases further TfR1-mediated iron internalisation, and favours the storage of excess intracellular iron into ferritin [1].

### Liver-Related Iron Metabolism Disorders

Primary or hereditary haemochromatosis (HH) is a disease characterised by iron overload and disproportionately low hepcidin levels. Many types of HH exist; type I results from mutations in HFE gene, type IIa is due to HJV gene mutations, and type III is due to TfR2 gene mutations. In all these cases, the liver synthesises less hepcidin, resulting in high ferroportin levels. This leads to increased intestinal iron absorption, low iron levels in RES macrophages, transferrin saturation, and the appearance of reactive NTBI in blood. Hepatic iron overload occurs, and, as previously mentioned, the liver is vulnerable in this state to damage, necrosis, and fibrosis [18]. There is also HH type IV ferroportin disease, which is an autosomal dominant hereditary iron loading disorder associated with heterozygote mutations of the ferroportin-1 (FPN) gene.

Secondary haemochromatosis is characterised by iron overload secondary to other medical conditions. For example, thalassaemia major and aplastic anaemia are conditions that require chronic transfusion therapies. Repeated transfusions lead to iron overload, and excessive iron storage can cause damage in iron-storing organs such as the liver and the heart. Furthermore, chronic liver disease due to alcoholism or viral hepatitis can impair hepatic hepcidin synthesis. The decreased hepcidin levels promote enhanced intestinal iron absorption, which leads to iron overload that can cause further hepatic damage [2].

## 6. Management

Table 1 presents some iron metabolism disorders, the common features associated with them, as well as how these disorders are managed [8,18].

## 7. Conclusions

Iron is an essential micronutrient for humans, as it is required in several vital biological processes. The fact that no active mechanism of iron excretion exists highlights the significance of this metal and possibly suggests that prehistoric humans did not have a constant, adequate iron intake. Iron metabolism is one of the most complex processes in the human body, involving many organs and tissues, such as the intestine, the bone marrow, the spleen, and the liver. Maintenance of iron homeostasis is under the influence of many proteins, with hepcidin being the key regulator. This liver-derived hormone regulates systemic iron levels through a negative feedback mechanism, and its synthesis is controlled by factors such as iron levels, anaemia, infection, inflammation, and erythropoietic activity. Cellular iron balance mechanisms also exist, and these include the interaction between IRPs and IREs. Physiological disorders within the different tissues involved in iron metabolism interfere with the mechanisms regulating iron homeostasis. These can ultimately result in iron deficiency or excess, both of which have detrimental effects on the organism. Labile iron and inflammatory conditions during infection may generate high levels of toxic free radicals and oxidative stress that can trigger cellular damage and cause diseases in different tissues and organs. Nowadays, research is increasingly dedicated in the field of iron metabolism, but countless questions remain unanswered. An increased knowledge of the physiology of iron homeostasis will facilitate understanding of the pathology of iron disorders and help improve treatment

## Figures and Tables

**Table 1:** Management of some disorders and diseases associated with iron.

Disorder	Features and Symptoms	Management
Peptic ulcers	Abdominal pain, nausea, vomiting, loss of appetite, weight loss, gastric/duodenal bleeding, anaemia.	Treatment of the underlying condition; iron supplementation; blood transfusion in severe cases.
Inflammatory Bowel Disease	Diarrhoea, low-grade fever, abdominal pain, blood in the stool, reduced appetite, weight loss, anaemia.	Corticosteroids, immunosuppressants.
Hookworm infection	Abdominal pain, fever, nausea, blood in the stool, anaemia.	Antihelminthic drugs (mebendazole); iron supplementation if anaemic.
Malabsorption syndromes	Diarrhoea, steatorrhoea, weight loss and fatigue, oedema, anaemia.	Gluten-free diet; iron and vitamin supplementation.
Thalassaemia syndromes	Skin pallor, jaundice, slowed growth, splenomegaly, facial bone deformities; transfusion-dependent iron overload.	Blood transfusions; iron chelation (deferoxamine, deferasirox) for secondary haemochromatosis; folic acid supplementation.
Sickle cell anaemia	Sickle cell crisis (abdominal pain, bone pain); anaemia; pallor, jaundice, hepatomegaly, splenomegaly in children followed by splenic infarction later, chronic leg ulcers; prone to pneumococcal septicaemia.	Prophylactic management (avoid precipitating factors); folic acid supplementation; in case of crisis: rehydration, antibiotics, and bicarbonate if acidotic; blood transfusion only if severe anaemia.
Sideroblastic anaemia	Skin pallor, fatigue, dizziness, splenomegaly; transfusion-dependent iron overload.	High-dose pyridoxine, folic acid supplementation, blood transfusion if severe anaemia; iron chelation (deferoxamine) if transfusion-related iron overload.
Anaemia of chronic disease	Tiredness, weakness, dyspnoea, skin pallor, dizziness.	Treatment of underlying cause if possible; iron supplementation is not beneficial; blood transfusion in severe anaemia.
Haemochromatosis	Joint pain, abdominal pain, fatigue, weakness, impotence; cirrhosis, diabetes mellitus, skin hyperpigmentation, heart failure.	Therapeutic phlebotomy; iron chelation therapy.
